# 

**Published:** 1891-05

**Authors:** 


### Society Notes.

Dr. Charles A. L. Reed, of Cincinnati, Chairman of the Section of Diseases of Women in the American Medical Association, has written a letter to the Journal of the Association, in which he advocates the organization of a Pan-American Medical Congress. He will present the subject at the meeting of the Association to be held in Washington, May 5–8, 1891, and hopes to have it there endorsed, and the machinery put in operation to perfect the scheme. In view of the recent legislation looking to a more intimate commercial relation between the United States and the South American republics, we are of the opinion that the proposition of Dr. Reed should be given serious consideration by the Association.

---

THE MEDICAL ASSOCIATION OF CENTRAL NEW YORK will hold its next annual meeting in Buffalo, June 2, 1891. Arrangements are in progress looking to an interesting and profitable meeting. As this is the first time the Association has visited Buffalo, it is hoped that an effort will be made by the local profession to attend and contribute to its success.

### BOOKS AND PAMPHLETS RECEIVED.

Sanders' Question Compends No. 2. Essentials of Surgery, together with a full description of the Handkerchief and Roller Bandage. Arranged in the form of questions and answers; prepared especially for students of medicine. By Edward Martin, A. M., M. D., Instructor in Operative Surgery, University of Pennsylvania; Surgeon to the Howard Hospital; Assistant Surgeon to the University Hospital. Illustrated; fourth edition, revised and enlarged by an appendix, containing full directions and prescriptions for the preparation of the various materials used in Antiseptic Surgery. Also several hundred receipts covering the medical treatment of surgical affections. Philadelphia: W. B. Sanders. 1891. Price, $1.00.

Koch's Remedy in Relation specially to Throat Consumption, By Lennox Browne, F. R. C. S. Ed., Senior Surgeon to the Central London Throat, Nose, and Ear Hospital; author of "The Throat and Nose, and their Diseases," etc. Illustrated by thirty-one cases and by fifty original engravings and diagrams. Philadelphia: Lea Brothers & Co. 1891.

Surgery: A Practical Treatise with Special Reference to Treatment. By C. W. Mansell Moullin, M. A., M. D., Oxon. Fellow of the Royal College of Surgeons; Surgeon and Lecturer on Physiology to the London Hospital; Formerly Radcliffe Traveling Fellow and Fellow of Pembroke College, Oxford, England. Assisted by various writers on special subjects. With 500 Illustrations, 200 of which have been made for this work from special drawings. 8vo pp. viii.—1180. Philadelphia: P. Blakiston, Son & Co. 1891. Buffalo: Peter Paul & Brother.

A Text-Book of Bacteriology. By Carl Fraenkel, M. D., Professor of Hygiene, University of Königsberg. Third edition; translated and edited by J. H. Linsley, M. D., Professor of Pathology and Bacteriology, Medical Department of the University of Vermont; Demonstrator of Pathology and Bacteriology, New York Post-Graduate Medical School and Hospital, etc., etc. Octavo, 380 pages. Extra muslin, $3.75. New York: William Wood & Company.

Practical Treatise on Electricity in Gynecology. By Egbert H. Grandin, M. D., Chairman Section on Obstetrics and Gynecology, New York Academy of Medicine; Obstetric Surgeon, New York Maternity Hospital; Obstetrician, New York Infant Asylum, etc., and Josephus H. Gunning, M. D., Instructor in Electro-Therapeutics, New York Post-Graduate Medical School and Hospital; Gynecologists to Riverview

Rest for Women ; Electro-Gynecologist, North-Eastern Dispensary, etc. Illustrated. Octavo, 180 pages. Muslin, $2.00. New York : William Wood & Company.

Diseases of the Digestive Organs in Infancy and Childhood, with chapters on the Investigation of Disease ; the Diet and General Management of Children, and Massage in Pediatrics. By Louis Starr, M. D., Late Clinical Professor of Diseases of Children in the Hospital of the University of Pennsylvania, etc. Second edition ; illustrated. Philadelphia : P. Blakiston, Son & Co. 1891.

Diabetes : Its Causes, Symptoms, and Treatment. By Charles W. Purdy, M. D., Queen's University ; Honorary Fellow Royal College of Physicians and Surgeons, Kingston ; Member of the College of Physicians and Surgeons of Ontario, etc. No. 8 in the Physicians' and Students' Ready Reference Series. With Clinical Illustrations. Philadelphia and London : F. A. Davis. 1890. Price, $1.25.

The Daughter, Her Health, Education, and Wedlock. Homely Suggestions for Mothers and Daughters. By William Capp, M. D. Philadelphia and London : F. A. Davis. 1891. Price, $1.00, net.

Manual of the Domestic Hygiene of the Child. For the use of Students, Physicians, Sanitary Officials, Teachers and Mothers. By Julius Uffiemann, M. D., Professor of Internal Medicine at the University of Rostock. Translated, with the author's kind permission, by Harriot Ransom Wilinowski. Edited by Mary Putnam Jacobi, M. D. Octavo, pp. x.—229. New York and London : G. P. Putnam's Sons. *The Knickerbocker Press.* 1891.

A Compend of Gynecology (Quiz-Compends, No. 7). By Henry Morris, M. D., Late Demonstrator of Obstetrics and Diseases of Women and Children in the Jefferson Medical College, Philadelphia. With forty-one illustrations. Philadelphia : P. Blakiston, Son & Co. 1891.

Electricity : Its Application on Medicine and Surgery. A Brief and Practical Exposition of Modern Scientific Electro-Therapeutics. By Wellington Adams, M. D. In two volumes. 12mo. Physicians' Leisure Library. Detroit : George S. Davis. 1891. Price, cloth, 50 cents ; paper, 25 cents.

Taking Cold. By Francke H. Bosworth, M. D., Professor of Diseases of the Throat in the Bellevue Hospital Medical College of New York. Physicians' Leisure Library. Detroit : George S. Davis. 1891. Price, cloth, 50 cents ; paper, 25 cents.

King's Journal Directory and Buyer's Guide. For the Convenience of Publishers, Advertisers, Manufacturers, etc., etc. Compiled and edited by Ferdinand King, Ph. Gr. Vol. II. New York : Dr. F. King, Publisher. 1891. Price, 50 cents.

Medical Education, Medical Colleges, and the Regulation of the Practice of Medicine in the United States and Canada. 1765—1891. Medical Education and the Regulation of the Practice of Medicine in Foreign Countries. (Illinois State Board of Health.) By John H. Rauch, M. D., Secretary. Springfield, Ill. : H. W. Rokker, State Printer and Binder. 1891.

The Professional Canvasser. No. 5. A Price-list of Stationery. By Fred. D. Van Horen, 265 Broadway, New York.

Posterior Displacements of the Uterus. Remarks upon Pathology and Treatment. By Isaac S. Stone, M. D., Washington, D. C. Reprint :

Transactions American Association of Obstetricians and Gynecologists. Volume III. 1890.

Inflammation in and about the Head of the Colon. By L. S. McMurtry, M. D., Gynecologist to Sts. Mary and Elizabeth Hospital; Fellow of the American Association of Obstetricians and Gynecologists, etc., Louisville, Ky. Reprint: *The Medical News*, January 10, 1891.

An Analysis of the Ocular Symptoms found in the Third Stage of General Paralysis of the Insane. By Charles A. Oliver, M. D., of Philadelphia. Reprint: *The Medical News*, September 20, 1890.

Annual Report of the Health Department of the City of Baltimore to the Mayor and City Council, for the year ending December 31, 1890. From George H. Rohé, M. D., Commissioner of Health and Registrar. Baltimore: John Cox, City Printer. 1891.

Cornell University, College of Agriculture. Third Annual Report of the Agricultural Experiment Station. Illustrated. Ithaca, N. Y.: Published by the University. 1891.

A Further Study of Anodal Diffusion as a Therapeutic Agent. By Frederick Paterson, M. D., Attending Physician to the New York Hospital for Nervous Diseases. Reprint: *The Medical Record*, January 31, 1891.

A Second Note upon Homonymous Hemiopic Hallucinations. Same author. Reprint: *The New York Medical Journal*, January 31, 1891.

Proceedings and Addresses at a Sanitary Convention held at Battle Creek, Mich., June 25-26, 1890. Supplement to the Report of the State Board of Health for 1890. Lansing: Robert Smith & Co., 1891.

A Case of Intra-cranial Neoplasm, with Localizing Eye Symptoms. Position of the Tumor verified at Autopsy. By Charles A. Oliver, M. D., Philadelphia. Reprint: *Archives of Ophthalmology*, Volume XX., No. 1, 1891.

Zehn weitere Fälle von Ventrofixatio Uteri Retroflexi. Von Dr. Sperling, früher zweitem Assistenzarzt an der Königl. Frauenklinik in Dresden, jetzt in Moabit bei Berlin. Mit einem Zusatz von Professor Dr. Leopold. Sonderabdruck aus der *Deutschen Medicinischen Wochenschrift*, 1891, No. 5. Redacteur: Geh. Sanitätsrath Dr. S. Guttmann. Leipzig: Verlag von Georg Thieme. 1891.

The Modern Antipyretics: Their Action in Health and Disease. By Isaac Ott, M. D., Consulting Physician to the Easton Hospital, Easton, Pa. 8vo, pp. 52. E. D. Vogel. 1891.

Bacteriology and Preventive Medicine. By Stephen Smith Burt, A. M., M. D., Professor of Clinical Medicine and Physical Diagnosis, New York Post-Graduate Medical School and Hospital, etc. Reprint: *The Post-Graduate*, April, 1891.

Intra-Peritoneal Myo-Fibroma of the Rectum, weighing twelve pounds, successfully removed by Laparatomy. By N. Senn, M. D., Ph. D. Reprint: *The Weekly Medical Review*, March 21, 1891.

Annual Message of Hon. Charles F. Bishop, Mayor of Buffalo. January, 1891. Buffalo: Times Printing House. 1891.

Atypic Herpes Zester Gangrenosa, with Report of two Cases. By Benj. Merrill Ricketts, Ph. B., M. D., Cincinnati, O. Reprint: *Journal of the American Medical Association*, Novamber 29, 1890.

The Radical Treatment of Hernia, with the Report of two Unique

Cases. By Benj. Merrill Ricketts, Ph. B., M. D., Cincinnati, O. The Surgical Treatment of Epilepsy. Same author. Reprint: Cincinnati *Lancet-Clinic*, January 24, 1891.

On the Employment of Women Physicians in Hospitals for the Insane. By Edward N. Brush, M. D., Philadelphia, Pa. Reprint: *American Journal of Insanity*, January, 1891.

Carlsbad, and the Natural Carlsbad Sprudel Salt in Powder form. The Eisner and Mendelson Company, sole agents, New York and Philadelphia.

Chronic Urethritis. By L. Bolton Bangs, M. D., Surgeon to St. Luke's and Charity Hospital, New York. Reprint: *The Therapeutic Gazette*, February 16, 1891.

Macroscopic Vocabulary of the Brain, with Synonyms and References, together with other reports. By Burt. G. Wilder, M. D., Professor of Comparative Anatomy, etc., in Cornell University.

A Case of Successful Trephining for Subdural Hemorrhage, produced by Contre-Coup. By John Homans, M. D., Visiting Surgeon to Massachusetts General Hospital; and George L. Walton, M. D., Physician to the Neurological Department of the same. Reprint: *Boston Medical and Surgical Journal*, February 12, 1891.

Reports of the Trustees and Superintendent of the Butler Hospital for the Insane, presented to the Corporation at its Forty-seventh Annual Meeting, January 28, 1891.

Sulfonal-Bayer. Recent Observations on its Value. William H. Schieffelin & Co., New York.

The Blood and Blood-Vessels, in Health and Disease. By Wesley Mills, M. A., M. D,, Professor of Physiology in McGill University, Montreal. Reprint: *The New York Medical Journal*, September 13, 1890.

Original Research in Relation to Animal Economics. A Socialistic Study. By Frank S. Billings, M. D. Reprint: *The Times and Register*, January 24, 1891.

The Johns Hopkins' Hospital Reports. Report in Surgery I. The Treatment of Wounds, with Especial Reference to the Value of the Blood-Clot in the Management of Dead Spaces. By William S. Halstead, M. D.

Le Courant Continu en Gynécologie.—Outillage.—Technique.—Effets Physiologiques. Publie par Le Docteur Georges Gautier. Paris. 1890. A. Maloine, éditeur.

Exposición de Varios Cases de Sifilis y de Algunas Anomalias Anatómicas del Aparato Genital de la Mujer. Por el Dr. D. Juan Soler y Buscallá. Barcelona. 1890.

A Dermatological Bibliography. Compiled by George Thomas Jackson, M. D., New York. 1891.

Vaginal Hysterectomy for Malignant Disease of the Uterus. By Florian Krug, M. D., New York. Reprint: *American Journal Obstetrics*, June, 1890.

Extirpation of Sarcomatous Ovaries in a Pseudo-Hermaphrodite. By Florian Krug, M. D., Gynecologist to the German Hospital, New York. Reprint: *American Journal Obstetrics*, January, 1891.

Transperitoneal Hysterorrhaphy: A new method of Ventral Fixation of the Uterus, without Opening the Peritoneal Cavity. By Florian Krug, M. D. Reprint: *The New York Medical Journal*, January 3, 1891.

Therapeutic Oxygen. By Samuel S. Wallian, A. M., M. D., of New York. Reprint: *The Medical News*, May 7, 1890.

Notes on the Examination for Tubercle Bacilli. By Ludwig Weiss, M. D. Reprint: *The New York Medical Journal*, January 17, 1891.

Thirteenth Annual Report of the Presbyterian Eye, Ear, and Throat Charity Hospital, Baltimore, Md.

Tonic Reconstituents. By A. Lutaud, M. D., Paris. Reprint: *Journal de Médicine de Paris*, January 18, 1891.

A Study of Sterility: Its Causes and Treatment. By Thomas W. Kay, M. D., Scranton, Pa. An Essay which received the first prize of the Alumni Association of the College of Physicians and Surgeons, Baltimore. Reprint: *Journal American Medical Association*, February 7, 14 and 21, 1891.

On the Dangers Arising from Syphilis in the Practice of Dentistry. By L. Duncan Bulkley, A. M., M. D., Attending Physician to the New York Skin and Cancer Hospital. Reprint: *International Dental Journal*, August and September, 1890.

Des Résultats Immédiats et Éloignés du Treatment Électrique des Fibromes Utérins, par la method du Docteur Apostoli. Par Mlle. Félicia Jakubowska, Docteur en Médecine de la Faculté de Paris. Paris: Octave Doin. 1890.

Charter, Constitution and By-Laws, List of Fellows, and Current Medical Periodicals on file in the Library of the New York Academy of Medicine. 1891.

The Rational Treatment of Uterine Displacements, based upon a Consideration of the Pathological Conditions Present. By Augustus S. Goelet, M. D., New York. Reprint: *American Journal Obstetrics*, February, 1891.

A Case of Epilepsy with Double Consciousness. By G. R. Trowbridge, M. D., Assistant Physician to the State Hospital for the Insane, Danville, Pa. Reprint: *The Medical News*, February 21, 1891.

Report of a Case of Large Interstitial Fibroid of the Uterus, Removed by Abdominal Section. By Thomas H. Manley, M. D., Visiting Physician to Harlem Hospital, New York. Reprint: *The Brooklyn Medical Journal*, February, 1891.

Resection of the Optic Nerve. By L. Webster Fox, Philadelphia. Reprint: *The Medical and Surgical Reporter*.

My Recent Experience in Operating for the Lacerations of the Perineum, involving the Sphincter Ani, with a description of my Method of Flapsplitting. By Horace Tracy Hanks, M. D. New York: Reprint Vol. XV. Transactions American Gynecological Society. 1890.

Deafness as a Result of Nasal and Dental Disease. By D. H. Goodwillie, M. D. Reprint: *The New York Medical Journal*, August 24, 1889.

A Death Caused by a Uterine Dilator. With some remarks as to the proper method of using the Dilator. By Howard A. Kelly, M. D. Reprint: *American Journal Obstetrics*, Vol. XXIV., No. 1. 1891.

Amygdalotomie et Hémorrage. Par le Docteur E. J. Moure, Professor libre des Maladies du Larynx, des Oreilles, et du Nez. Paris: Octave Doin. 1891.

A Submembraneous Local Treatment of Pharyngeal Diphtheria. By A. Seibert, M. D., New York. Reprint: The *New York Medical Journal*, December 6, 1890.

In What Class of Wounds shall We Use Drainage? By Henry Orlando Marcy, A. M., M. D., L. L. D. Boston: Reprinted from the Transactions American Association Obstetricians and Gynecologists. Vol. III. 1890.

The Surgical Treatment of Non-Pedunculated Abdominal Tumors. By Henry O. Marcy, M. D. Boston: Reprint: *The Journal American Medical Association*, November 8, 1890.

Surgical Relief for Biliary Obstruction. By Henry O. Marcy, M. D. Boston: Reprint: *The Journal American Medical Association*, December 20, 1890.

The Non-Operative Treatment of Delayed Union in Fractures of the Leg. By John Ridlon, M. D., New York. Reprint: The *Medical Record*, January 31, 1891.

The Treatment of Arterio-Venous Aneurism, with Two Cases Treated by Extirpation. By B. Farquhar Curtis, M. D., New York. Reprint: *The American Journal of the Medical Sciences*, February, 1891.

The Franklinic Interrupted Current, or my New System of Therapeutic Administration of Static Electricity. By William James Morton, M. D., New York. Reprint: *The Medical Record*, January 24, 1891.

Annual Report of the Erie County Penitentiary for the year ending September 30, 1890. Buffalo: George E. Matthews & Co. 1891.

On the Subjection of Consumption. By Wm. H. Dukeman, M. D., Los Angeles, Cal. Reprint: *The Pacific Medical Journal*.

Twenty-first Annual Report of the Buffalo Park Commissioners. January, 1891. Buffalo: Haas & Klein. 1891.

HEALTH BULLETINS.

Tennessee State Board of Health, February 20th, March 20th. and April 20, 1891.

Michigan State Board of Health, March, 1891.

New York State Board of Health, February and March, 1891.

Newport, R. I., Board of Health, February, March, 1891.

Abstract of Sanitary Reports, U. S. Marine Hospital Service. Vol. VI., Nos. 9, 10, 11, 12, 13, 14, 15, and 16, 1891.

Notice to Contributors. — We are glad to receive contributions from every one who knows anything of interest to the profession. Articles designed for publication in the Journal should be handed in before the first day of the month. The Editors are not responsible for the views or opinions of contributors. All communications should be addressed to 284 Franklin Street, Buffalo, N. Y.

